# Sleeve lobectomy versus pneumonectomy for non-small cell lung cancer: a meta-analysis

**DOI:** 10.1186/1477-7819-10-265

**Published:** 2012-12-11

**Authors:** Woda Shi, Wei Zhang, Haoliang Sun, Yongfeng Shao

**Affiliations:** 1 Department of Cardio-Thoracic Surgery, the third People’s hospital, Yancheng, China; 2 Department of Cardio-Thoracic Surgery, the first Affiliated Hospital of Nanjing Medical University, 300# guangzhou road, Nanjing, 210029, China

**Keywords:** Meta-analysis, Sleeve lobectomy, Pneumonectomy, Non-small cell lung cancer

## Abstract

**Aim:**

It is controversial that whether sleeve lobectomy (SL) should be promoted more worthy than pneumonectomy (PN) in suitable patients.

**Methods:**

We searched all studies that had been published in English from PUBMED and Embase which compared the short-term and long-term outcomes of SL and pneumonectomy (PN) in patients with non-small cell lung cancer (NSCLC).

**Results:**

Nineteen studies met our criteria with a combined total of 3878 subjects, of which 1316 (33.9%) underwent SL and 2562 (66.1%) underwent PN. The odds ratio was 0.50 (95% CI: 0.34-0.72) for postoperative mortality, 1.17 (95% CI: 0.82-1.67) for postoperative complications, 0.78 (95% CI: 0.47-1.29) for locoregional recurrences. The risk difference for 1-, 3-, 5- year was 0.11 (95% CI: 0.07-0.14), 0.15 (95% CI: 0.06-0.24), 0.15 (95% CI: 0.09-0.20),respectively. The pooled hazard ratio was 0.63 (95% CI: 0.56-0.71) in favor of SL group.

**Conclusion:**

SL is more worthy to be done than PN in suitable patients with less mortality and better long-term survival.

## Background

Since sleeve lobectomy uasge in lung cancer was first introduced by Sir Prince Thomas in 1947 [1], it has been regarded as standard management for treatment of patients with non-small cell lung cancer (NSCLC), who have low-grade, centrally located lesions and whose pulmonary reserve is insufficient and does allow pneumonectomy. As patient-selection criteria have changed and surgical techniques have been improved over the years, sleeve lobectomy is also performed for such patients who can tolerate pneumonectomy. Even when the tumors involve not only the airway but also the central vascular structures, in particular the pulmonary artery, sleeve lobectomy is done concomitantly with pulmonary artery reconstruction (double sleeve) in preference to pneumonectomy. Several studies have shown that long-term survival after sleeve lobectomy is similar to or even better than that after penumonectomy with better preservation of lung function, and a better quality of life. However, it remains controversial whether sleeve lobectomy is associated with a high rate of postoperative complications and operative mortality. In the current meta-analysis, we sought to assess whether sleeve lobectomy concomitant with or without pulmonary artery reconstruction or pneumonectomy offered a low morbidity and mortality and a better long-term survival for NSCLC patients.

## Methods

### Search strategy and study selection

Relevant studies were identified and selected by searching the databases-PubMed and Embase (updated to October 2011), -using the search words ‘sleeve resection or lobectomy and/or pneumonectomy’ and ‘lung neoplasm and/or non-small cell lung cancer and/or NSCLC)’. Written informed consent was obtained from the patient for publication of this report and any accompanying images. Studies on sleeve lobectomy concomitant with pulmonary artery reconstruction (double sleeve) were also searched using the search words ‘pulmonary artery reconstruction or pulmonary artery sleeve resection or bronchovascular sleeve resection’. We also scanned bibliographies in relevant articles and conference proceedings. The following selection criteria were applied: (1) non-small-cell lung cancer; (2) comparison of the surgical results of sleeve lobectomy concomitant with or without pulmonary artery reconstruction versus pneumonectomy; (3) the trials should report on pulmonary artery reconstruction associated with lobectomy or bronchial sleeve resection; (4) the trials should report at least one outcome; (5) a given patient population was used only once; if the same population appeared in other publications, the article that provided the most complete follow-up data was selected; (6) we excluded trials in which participants were subjected to carinal or tracheal resection.

### Data extraction

Data were independently extracted from each study by two researchers (SYF, SWD). Any disagreement was resolved by discussion and consensus opinion. We extracted data on study characteristics, patient clinical characteristics and demographics, histologic type of tumor, distribution of stage, and duration of follow-up. Primary outcomes included postoperative mortality, postoperative complications; additional outcomes included locoregional recurrences, difference of survival of the two techniques at 1, 3, and 5 years, and other adverse events.

### Statistical methods

Meta-analysis was carried out using odds ratio (OR), risk difference (RD), and hazard ratio (HR) as the primary effect measures. The effect measure OR was used to analyze the odds of an adverse event occurring in the sleeve lobectomy group compared to the pneumonectomy group, while RD was used to analyze the difference in survival of patients in the sleeve lobectomy and the pneumonectomy groups. A fixed-effects or random-effects model was employed [2].

For each meta-analysis result, Cochran’s Q and I2 statistics were first calculated to assess the heterogeneity among the proportions of the included trials. If the *P*- value was < 0.1, the assumption of homogeneity was deemed invalid, and the random-effects model was reported after exploring the causes of heterogeneity [3]. Otherwise, the fixed-effects model was reported. The cumulative meta-analysis [4] over time was also used for postoperative mortality and complications. If the OR was less than 1, while the RD was more than 0, this favored the sleeve lobectomy group, and the points of estimate of the OR and RD were considered statistically significant at the P < 0.05 level if the 95% confidence interval did not include the value 1 or 0, respectively. The log HR and its variance were used for the time-to-event analysis. In case the hazard ratio (HR) was not directly given in the publication, we extracted summary statistics from Kaplan-Meier product-limit estimations and estimated HRs according to methods proposed by Parmar in 1998 [5]. For estimation, we applied a tool, which uses P-values of the appropriate log-rank test comparing the two survival functions of interest, number of patients analyzed, and number of events in each arm [6]. If this information was not available, HR was deduced from the graphical display of the survival curves, if possible. Survival data were pooled by the Der Simonian and Laird method to produce a random-effect meta-analysis. Heterogeneity between studies was investigated by the standard chi-squared Q-test.

The effect of publication and selection bias on the summary estimates was tested by both the Harbord-Egger bias indicator and Begg-Mazumdar bias indicator. Also, a funnel plot was constructed to evaluate potential publication bias by using the standard error of the log OR and log OR. A two-tailed P-value < 0.05 was considered statistically significant. Calculation was conducted using STATA version 11.0 (Stata Corporation, College Station, TX, USA) and Microsoft Excel 2007 (Microsoft Corporation, USA).

## Results

### Selected studies

Our search yielded a total of 544 potentially relevant clinical studies on sleeve lobectomy versus pneumonectomy. After excluding review articles, observational studies, case reports, meta-analyses, a total of 19 trials [7-25] with 3,878 subjects were included in this analysis; these, 1,316 patients (33.9%) had undergone sleeve lobectomy and 2,562 (66.1%) pneumonectomy.

The characteristics of these studies are shown in Table 1. The distribution of stages in the sleeve lobectomy group and the pneumonectomy group was significantly different (stages I, II, and III, 35.00%, 38.32%, and 26.68% for sleeve lobectomy, and 19.72%, 32.32%, and 47.96% for pneumonectomy; P < 0.001). Sex ratios for the two groups showed no significant difference (male/female, 82.16%/17.84% for sleeve lobectomy, and 80.00%/20.00% for pneumonectomy; P = 0.134). There was no difference between the two groups in mean age (62.88 years for sleeve lobectomy, 62.06 years for pneumonectomy; P = 0.4963), although age distributions were not available.

**Table 1:** Study characteristics included in the meta-analysis

**Authors**	**Year**	**Number**		**Mean age**		**Male/female**		**Stage I**		**Stage II**		**≥ Stage III**	
		**SL**	**PN**	**SL**	**PN**	**SL**	**PN**	**SL**	**PN**	**SL**	**PN**	**SL**	**PN**
Gaissert et al.	1996	72	56	63.4	60.8	56/16	42/14	29	9	31	25	12	22
Yoshino et al.^a^	1997	29	29	60.6	58.2	26/3	23/6	9	9	12	12	8	8
Suen et al.^b^	1999	58	142	63.7	66.5	41/17	81/61	18	37	28	46	12	59
Okada et al.	2000	60	60	60.9	60.6	52/8	53/7	/	/	/	/	/	/
Ghiribelli et al.	2002	38	127	65	62.4	36/2	102/25	16	29	10	43	12	55
Martin et al.	2002	38	81	65	63	27/11	63/18	10	10	16	32	12	39
Deslauriers et al.	2004	184	1,046	60	60.7	152/32	827/219	82	164	72	361	30	521
Bagan et al.	2005	66	151	60.7	58.16	58/8	138/13	40	35	14	35	12	81
Kim et al.	2005	49	49	58.7	58.1	44/5	46/3	14	24	20	13	15	12
Lausberg et al.	2005	171	63	61.5	60.9	136/35	56/7	33	7	80	32	58	24
Ludwig et al.	2005	116	194	62	59	/	/	31	32	41	52	44	110
Takeda et al.	2006	62	110	61.1	59.3	46/16	92/18	26	24	19	14	17	72
Balduyck et al.	2008	10	20	65.3	63.3	/	/	2	3	1	9	7	8
Melloul et al.	2008	69	78	/	/	/	/	15	28	30	21	24	29
Parissis et al.	2009	79	129	60.44	62.5	54/25	91/38	/	/	/	/	/	/
Hanagiri et al.^c^	2010	24	72	65.1	64.7	18/6	61/11	5	5	8	13	11	54
Park et al.	2010	105	105	61.25	62.24	99/6	98/7	44	43	32	36	29	26
Bolukbas et al.	2011	31	29	73.6	74.2	25/6	25/4	5	2	17	10	9	17
Gomez-Caro et al.	2011	55	21	63.5	62.4	51/4	18/3	33	7	20	13	2	1
Total		1,316	2,562	62.89	62.06	921/200	1,816/454	412	468	451	767	314	1,138

^a^Containing two cases of small-cell carcinoma in the sleeve lobectomy (SL) group and one in the pneumonectomy (PN) group; ^b^containing two cases of resection of the tracheal carina in SL; ^c^containing two cases of right upper sleeve lobectomy with carinoplasty in SL.

### Postoperative mortality

**Figure 1:**
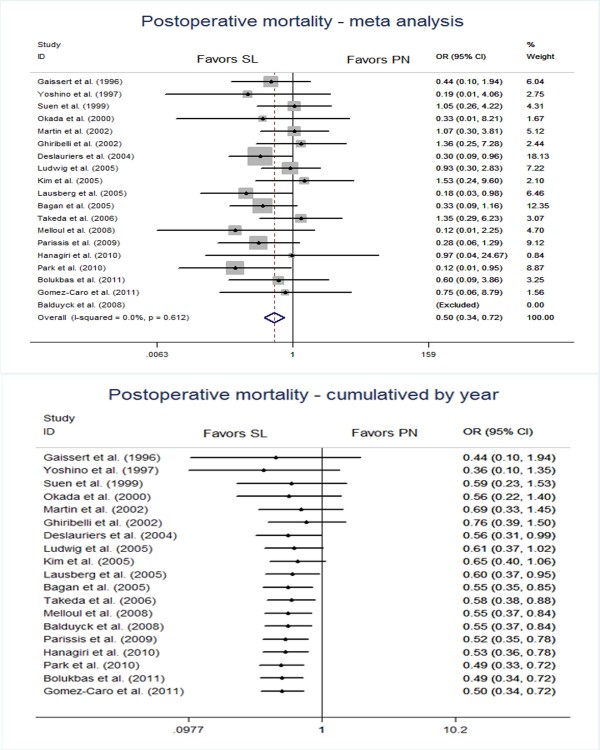
Conventional and cumulative meta-analysis of selected studies comparing postoperative mortality between the sleeve lobectomy group and the pneumonectomy group.

The meta-analysis showed that the pooled postoperative mortality in patients undergoing sleeve lobectomy was 2.91% (38/1,306) as compared with 5.86% (149/2,542) in patients receiving pneumonectomy, and there was a significant difference in the postoperative mortality, which favored the sleeve lobectomy group (OR 0.50, 95% CI 0.34, 0.72), as shown in Figure 1. By plotting the emergence of sleeve lobectomy with time (Figure 1), it was clear that the earlier trials of sleeve lobectomy fitting our inclusion criteria conducted before 2005 demonstrated a high degree of heterogeneity. Since 2005, the overall effect size for postoperative mortality has remained relatively stable within an effect size between OR of 0.49 and 0.60.

### Postoperative complications

**Figure 2:**
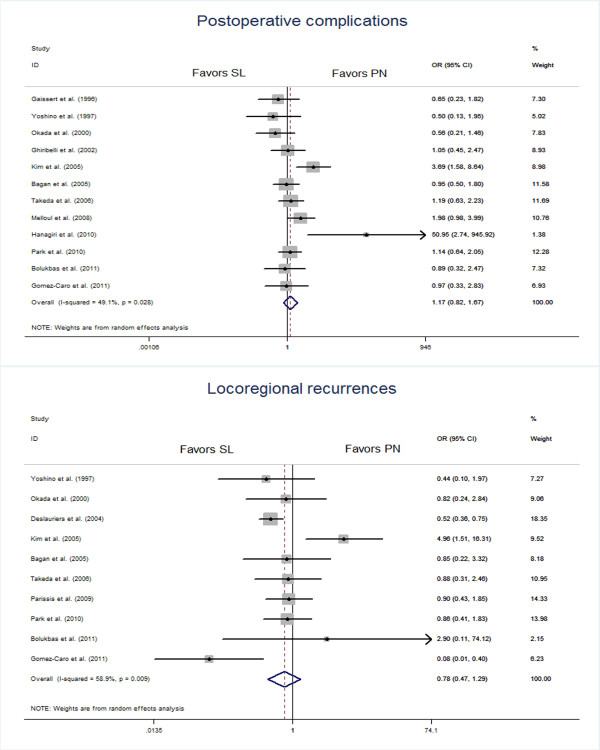
Meta-analysis of selected studies comparing postoperative complications and locoregional recurrences between the sleeve lobectomy group and the pneumonectomy group.

Twelve studies [7,8,10,11,14,15,18,20,22-25] reported the incidence of postoperative complications, and the meta-analysis showed a pooled incidence of 32.88% (217/660) with sleeve lobectomy, and 27.06% (240/887) with pneumonectomy (OR 1.17, 95% CI 0.82, 1.67), but this was not statistically significant (Figure 2). The cumulative meta-analysis also showed no statistical significance.

### Locoregional recurrences

Locoregional recurrences were reported in ten studies [8,10,13-15,18,21,23-25] and resultant data from meta-analysis showed the pooled locoregional recurrence in sleeve lobectomy was 14.44% (104/720) compared with 26.08% (451/1,729) in pneumonectomy, but this was not statistical significant (OR 0.78, 95% CI 0.47, 1.29), as shown in Figure 2.

### Survival at 1, 3, and 5 years and overall survival

**Figure 3:**
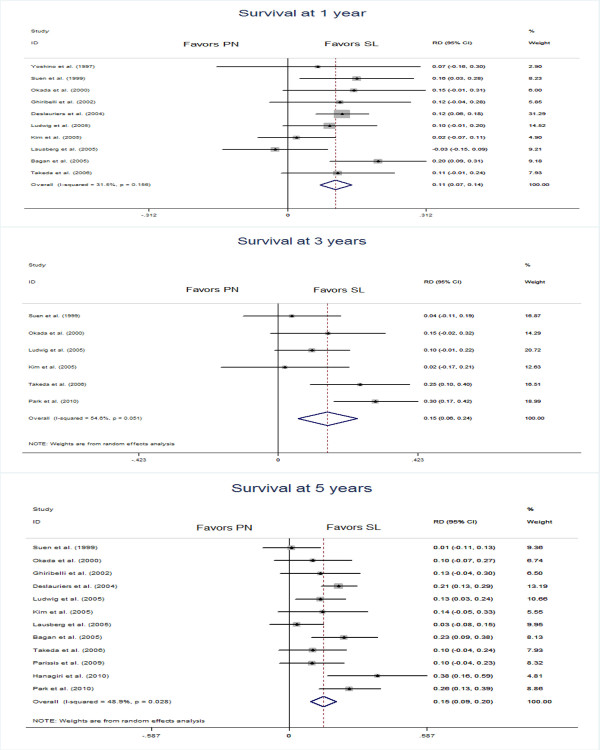
Meta-analysis of selected studies comparing differences in survival at 1, 3, and 5 years between the sleeve lobectomy group and the pneumonectomy group.

**Figure 4:**
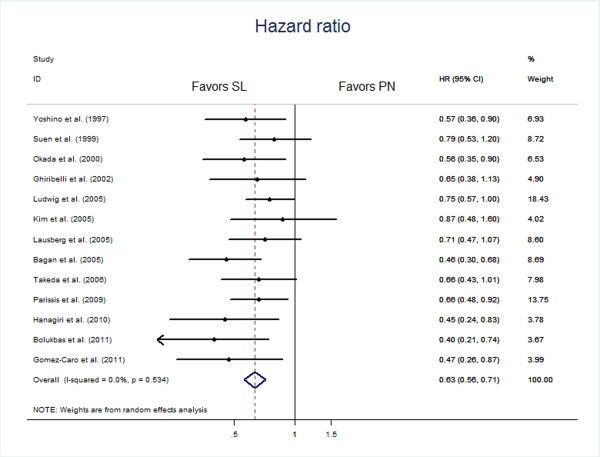
Meta-analysis of overall survival hazard ratios in individual studies and across all studies.

Ten studies [8-11,13-18] were extracted for analysis of the differences of survival at 1 year, which showed a combined RD of 0.11 (95% CI 0.07, 0.14) in favor of the sleeve lobecteomy group (Figure 3). Similar results were achieved for differences in survival at 3 years (in six studies [9,10,15,17,18,23], RD 0.15, 95% CI 0.06, 0.24) and 5 years (in twelve studies [9-11,13-18,21-23], RD 0.15, 95% CI 0.09, 0.20). All the estimated combined RD were statistically significant. In addition, 13 studies [8-11,14-18,21,22,24,25] including 2,014 patients, 838 in the sleeve lobectomy group and 1,176 in the pneumonectomy group were extracted for meta-analysis of overall survival. The summaries of individual studies and overall pooled survival are shown in Figure 4. The estimated combined HR for overall survival in 13 studies was 0.63 (95% CI 0.56, 0.71) in favor of the sleeve lobectomy group, and there was a statistically significant difference.

### Publication bias

**Figure 5:**
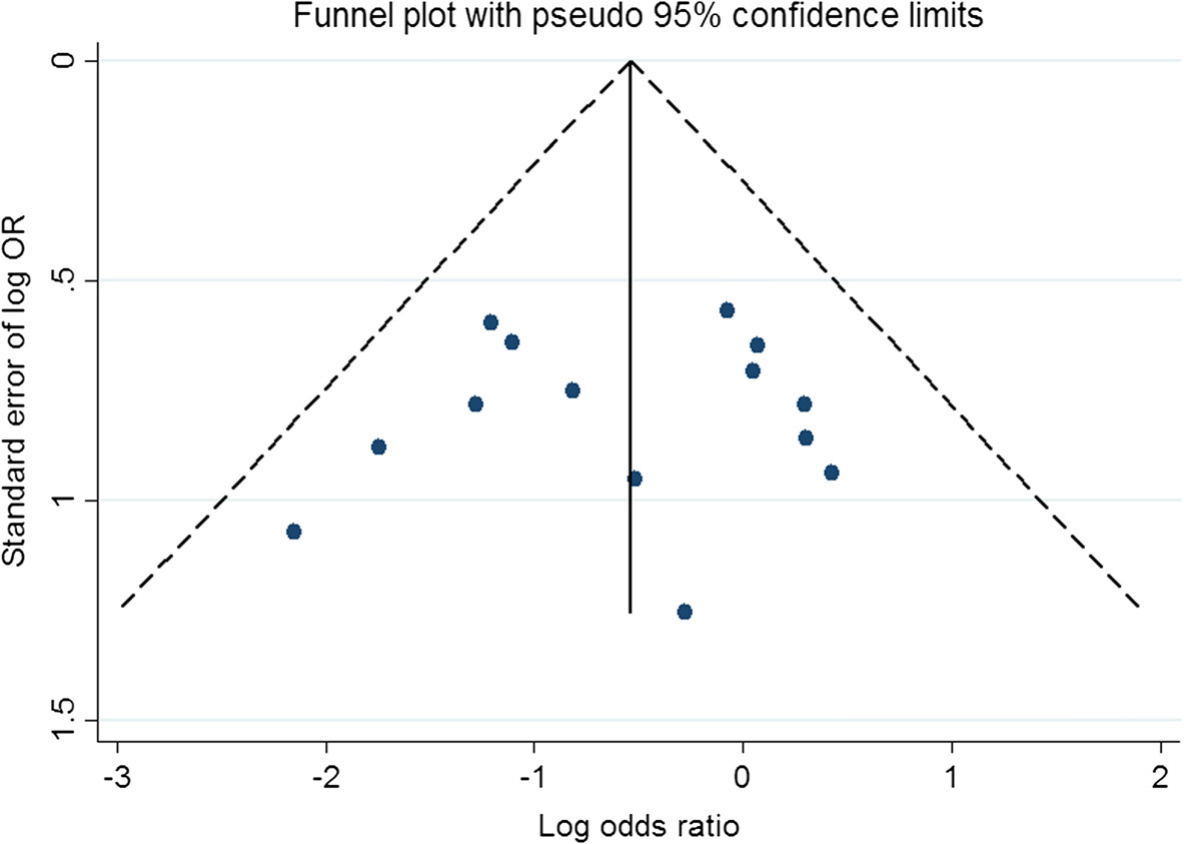
Funnel plot evaluating the effect of publication bias on studies for postoperative mortality.

Presence of publication bias analysed using the Harbord-Egger bias indicator gave a value of -0.42 (95% CI -1.51-, 1.01, *P* = 0.68), indicating that there was no publication bias. The Begg-Mazumdar indicator gave a Kendall tau b value of 0.11 (*P* = 0.91), suggesting no publication bias. The funnel plots also show no publication bias (Figure 5).

## Discussion and conclusion

Our study analyzed 19 clinical trials of sleeve lobectomy versus pneumonectomy, including a total of 3,878 subjects, of whom 1,316 (33.9%) underwent sleeve lobectomy and 2,562 (66.1%) underwent pneumonectomy. The sex ratios or mean ages for the two groups showed there were no significant difference, but the distribution of stages in the sleeve lobectomy group and pneumonectomy groups was significantly different (stages I, II, and III: 35.00%, 38.32%, and 26.68% for sleeve lobectomy; 19.72%, 32.32%, and 47.96% for pneumonectomy; P < 0.001; 17 reports), indicating that sleeve lobectomy is promoted as more worthy for early stage NSCLC and may has a better prognosis.

Patients receiving sleeve lobectomy showed significant superior outcomes compared to those receiving pneumonectomy in terms of postoperative mortality, especially for trials after 2005, indicating that sleeve lobectomy is more likely to be performed with advances in surgical technology and skills. In terms of postoperative complications and locoregional recurrences, no significant difference was found between patients receiving sleeve lobectomy and those receiving pneumonectomy. Patients who had undergone sleeve lobectomy showed a clear advantage over those who received pneumonectomy in terms of 1, 3, and 5-year survival and the time-to-event. We also tried to carry out meta-analysis of differences in long-term survival by different clinical stages or nodal status between patients receiving sleeve lobectomy and pneumonectomy, but we failed to obtain an adequate number of eligible trials or sufficient clinical data.

Only five studies [7,13,17,18,21] compared the long-term survival between groups by different clinical stages, and six studies [10,11,13,15,17,21] by different nodal status. Both Deslauriers et al [13] and Okada, *et al*. [10] reported a better prognosis after sleeve lobectomy treatment in patients with stages I and II diseases. Takeda, *et al*. [18] did not report any difference in five-year survival for patients at stages I and II after sleeve lobectomy or pneumonectomy, but the overall five-year survival in the sleeve lobectomy group was better than in the pneumonectomy group (54% vs. 33%). Okada, *et al*. [10] reported a significant difference among patients classification of nodal disease (N) 0 or N1 in favor of sleeve lobectomy, and Deslauriers, *et al*. [13] reported a significant difference among patients with N0 disease in favor sleeve lobectomy. Both Okada, *et al*. [10] and Deslauriers, *et al*. [13] reported there was no significant difference among patients with N2 disease. Additionally, both Kim, *et al*. [15] and Parissis, *et al*. [21] reported there was no significant difference among patients with advanced nodal disease. Furthermore, Takeda, *et al*. [18] reported that patients with stage III cancer in the pneumonectomy group, who received induction therapy, had a marginally better survival rate compared to those in the sleeve lobectomy group.

Few studies compared the lung function injury [7,12,24,25] and quality of life [19,25] after the two operation procedures, and the evaluation indexes varied, which made us unable to perform a meta-analysis of these two outcomes. Martin, *et al*. [12] and Gomez-Caro, *et al*. [25] reported there was a significant difference in favor of those receiving sleeve lobectomy in mean perioperative loss of FEV1 (forced expiratory volume in one second) and FVC (forced vital capacity). Melloul, *et al*. [20] reported that the postoperative loss of FEV1 and DLCO (diffusing capacity for carbon monoxide) were significantly higher after pneumonectomy than after sleeve lobectomy in patients < 70 years of age, and that the postoperative loss of FEV1, but not DLCO (No statistical difference), was significantly higher after pneumonectomy than after sleeve lobectomy in patients > 70 years of age. Balduyck, *et al*. [19] reported there was a significant differences in physical functioning, role functioning, cognitive functioning and shoulder dysfunction in favor of sleeve lobectomy.

The limitations of the present meta-analysis were as follows: (1), some studies contained a few patients who did not have NSCLC or tracheal carina resection, as illustrated in Table 1. (2) The disease stage distribution in the sleeve lobectomy and pneumonectomy groups were quite different, which might have led to unreliable results and favored the sleeve lobectomy group. (3) The definitions of postoperative mortality, postoperative complications and locoregional recurrences were different. (4) Preoperative neoadjuvant therapy, and postoperative radiotherapy or chemotherapy may interfere with the survival results. (5) Most of studies were retrospective because a randomized prospective trial is not possible. (6) Publication bias might affect the meta-analytic results, potentially producing overstated conclusions. (7) The analysis of long-term results according to different stages and nodal status were dropped due to an insufficient number of studies and inadequate clinical data. Finally, there were not enough studies comparing the loss of lung function and quality of life after the two operation procedures.

In conclusion, our study demonstrates that, (1) with advances in patient selection criteria and surgical techniques, sleeve lobectomy with or without pulmonary artery reconstruction is effective and can be done safely with lower mortality and without increasing morbidity and locoregional recurrence, as compared to pneumonectomy. (2) Sleeve lobectomy could offer better long-term survival than pneumonectomy. (3) Patients receiving sleeve lobectomy may have less loss of function and better quality of life than patients receiving pneumonectomy, but more evidence is required.

## Competing interests

The authors declare that they have no competing interests.

## Authors’ contributions

Woda Shi and Yongfeng Shao drafted the manuscript. Woda Shi searched the papers and extracted the data. Wei Zhang and Haoliang Sun helped to extracted the data. All authors read and approved the final manuscript.

## References

[B1] Thomas CP Conservative resection of the bronchial tree J R Coll Surg Edinb 1956 1 169 186 13307666

[B2] DerSimonian R Laird N Meta-analysis in clinical trials Control Clin Trials 1986 7 177 188 10.1016/0197-2456(86)90046-2 3802833

[B3] Lau J Ioannidis JP Schmid CH Quantitative synthesis in systematic reviews Ann Intern Med 1997 127 820 826 9382404 10.7326/0003-4819-127-9-199711010-00008

[B4] Lau J Antman EM Jimenez-Silva J Kupelnick B Mosteller F Chalmers TC Cumulative meta-analysis of therapeutic trials for myocardial infarction N Engl J Med 1992 327 248 254 10.1056/NEJM199207233270406 1614465

[B5] Parmar MK Torri V Stewart L Extracting summary statistics to perform meta-analyses of the published literature for survival endpoints Stat Med 1998 17 2815 2834 10.1002/(SICI)1097-0258(19981230)17:24<2815::AID-SIM110>3.0.CO;2-8 9921604

[B6] Tierney JF Stewart LA Ghersi D Burdett S Sydes MR Practical methods for incorporating summary time-to-event data into meta-analysis Trials 2007 8 16 10.1186/1745-6215-8-16 17555582 PMC1920534

[B7] Gaissert HA Mathisen DJ Moncure AC Hilgenberg AD Grillo HC Wain JC Survival and function after sleeve lobectomy for lung cancer J Thorac Cardiovasc Surg 1996 111 948 953 10.1016/S0022-5223(96)70369-0 8622318

[B8] Yoshino I Yokoyama H Yano T Ueda T Takai E Mizutani K Asoh H Ichinose Y Comparison of the surgical results of lobectomy with bronchoplasty and pneumonectomy for lung cancer J Surg Oncol 1997 64 32 35 10.1002/(SICI)1096-9098(199701)64:1<32::AID-JSO7>3.0.CO;2-Q 9040798

[B9] Suen HC Meyers BF Guthrie T Pohl MS Sundaresan S Roper CL Cooper JD Patterson GA Favorable results after sleeve lobectomy or bronchoplasty for bronchial malignancies Ann Thorac Surg 1999 67 1557 1562 10.1016/S0003-4975(99)00372-0 10391254

[B10] Okada M Yamagishi H Satake S Matsuoka H Miyamoto Y Yoshimura M Tsubota N Survival related to lymph node involvement in lung cancer after sleeve lobectomy compared with pneumonectomy J Thorac Cardiovasc Surg 2000 119 814 819 10.1016/S0022-5223(00)70018-3 10733774

[B11] Ghiribelli C Voltolini L Luzzi L Paladini P Campione A Gotti G Survival after bronchoplastic lobectomy for non small cell lung cancer compared with pneumonectomy according to nodal status J Cardiovasc Surg (Torino) 2002 43 103 108 11803340

[B12] Martin-Ucar AE Chaudhuri N Edwards JG Waller DA Can pneumonectomy for non-small cell lung cancer be avoided? An audit of parenchymal sparing lung surgery Eur J Cardiothorac Surg 2002 21 601 605 10.1016/S1010-7940(02)00028-3 11932153

[B13] Deslauriers J Gregoire J Jacques LF Piraux M Guojin L Lacasse Y Sleeve lobectomy versus pneumonectomy for lung cancer: a comparative analysis of survival and sites or recurrences Ann Thorac Surg 2004 77 1152 1156 Discussion 1156 10.1016/j.athoracsur.2003.07.040 15063224

[B14] Bagan P Berna P Pereira JC Le Pimpec Barthes F Dujon A Foucault C Dujon A Riquet M Sleeve lobectomy versus pneumonectomy: tumor characteristics and comparative analysis of feasibility and results Ann Thorac Surg 2005 80 2046 2050 10.1016/j.athoracsur.2005.06.045 16305842

[B15] Kim YT Kang CH Sung SW Kim JH Local control of disease related to lymph node involvement in non-small cell lung cancer after sleeve lobectomy compared with pneumonectomy Ann Thorac Surg 2005 79 1153 1161 10.1016/j.athoracsur.2004.09.011 15797043

[B16] Lausberg HF Graeter TP Tscholl D Wendler O Schafers HJ Bronchovascular versus bronchial sleeve resection for central lung tumors Ann Thorac Surg 2005 79 1147 1152 10.1016/j.athoracsur.2004.09.009 15797042

[B17] Ludwig C Stoelben E Olschewski M Hasse J Comparison of morbidity, 30-day mortality, and long-term survival after pneumonectomy and sleeve lobectomy for non-small cell lung carcinoma Ann Thorac Surg 2005 79 968 973 10.1016/j.athoracsur.2004.08.062 15734415

[B18] Takeda S Maeda H Koma M Matsubara Y Sawabata N Inoue M Tokunaga T Ohta M Comparison of surgical results after pneumonectomy and sleeve lobectomy for non-small cell lung cancer: trends over time and 20-year institutional experience Eur J Cardiothorac Surg 2006 29 276 280 10.1016/j.ejcts.2005.12.017 16434204

[B19] Balduyck B Hendriks J Lauwers P Van Schil P Quality of life after lung cancer surgery: a prospective pilot study comparing bronchial sleeve lobectomy with pneumonectomy J Thorac Oncol 2008 3 604 608 10.1097/JTO.0b013e318170fca4 18520798

[B20] Melloul E Egger B Krueger T Cheng C Mithieux F Ruffieux C Ris HB Magnusson L Mortality, complications and loss of pulmonary function after pneumonectomy vs. sleeve lobectomy in patients younger and older than 70years Interact Cardiovasc Thorac Surg 2008 7 986 989 10.1510/icvts.2008.182279 18603544

[B21] Parissis H Leotsinidis M Hughes A McGovern E Luke D Young V Comparative analysis and outcomes of sleeve resection versus pneumonectomy Asian Cardiovasc Thorac Ann 2009 17 175 182 19592550 10.1177/0218492309103309

[B22] Hanagiri T Baba T Ichiki Y Yasuda M Sugaya M Ono K Uramoto H Takenoyama M Yasumoto K Sleeve lobectomy for patients with non-small cell lung cancer Int J Surg 2010 8 39 43 10.1016/j.ijsu.2009.10.004 19850158

[B23] Park JS Yang HC Kim HK Kim K Shim YM Choi YS Kim J Sleeve lobectomy as an alternative procedure to pneumonectomy for non-small cell lung cancer J Thorac Oncol 2010 5 517 520 10.1097/JTO.0b013e3181d0a44b 20104190

[B24] Bolukbas S Eberlein MH Schirren J Pneumonectomy vs. sleeve resection for non-small cell lung carcinoma in the elderly: analysis of short-term and long-term results Thorac Cardiovasc Surg 2011 59 142 147 10.1055/s-0030-1250426 21480133

[B25] Gomez-Caro A Garcia S Reguart N Cladellas E Arguis P Sanchez M Gimferrer JM Determining the appropriate sleeve lobectomy versus pneumonectomy ratio in central non-small cell lung cancer patients: an audit of an aggressive policy of pneumonectomy avoidance Eur J Cardiothorac Surg 2011 39 352 359 10.1016/j.ejcts.2010.07.002 21185734

